# Special Issue “Molecular Advances in Oncological Photodynamic Therapy”

**DOI:** 10.3390/ijms27052136

**Published:** 2026-02-25

**Authors:** Michal Heger

**Affiliations:** 1 Applive Research Laboratories, College of Medicine, Jiaxing University, Jiaxing 314001, China; michal.heger@photonanomedicine.com or m.heger@uu.nl; 2 Jiaxing Key Laboratory for Photonanomedicine and Experimental Therapeutics, Department of Pharmaceutics, College of Medicine, Jiaxing University, Jiaxing 314001, China; 3 Molecular Biophysics and Biochemistry, Department of Chemistry, Faculty of Science, Utrecht University, 3584 CH Utrecht, The Netherlands

Photodynamic therapy (PDT) is conceptually and operationally a relatively straightforward treatment paradigm. A photosensitizer (PS) is delivered to malignant tissue, light is applied to target tissue in a spectrally appropriate manner, and the ensuing photochemistry yields reactive oxygen species (ROS) that damage critical biomolecular targets [1,2,3,4,5,6,7]. This in turn triggers direct tumor eradication owing to spatially confined hyperoxidative stress and indirect (latent) tumor clean-up through immunological cell death and abscopal effects [4,8,9,10,11,12,13,14,15,16]. The efficacy of PDT is contingent on tightly linked elements: PS pharmacokinetics and intracellular localization, light delivery and dosimetry, oxygen availability, and the tumor microenvironment [3,4,6,17,18,19,20]. In practice, each element introduces its own failure modes and compensatory biology that lead to mechanistic misalignment at the expense of therapeutic efficacy [18,21,22,23,24,25,26,27,28]. It is therefore not surprising that molecular advances in oncological PDT increasingly arise at disciplinary interfaces: where tumor biology meets PS delivery engineering, where photophysics meets cellular stress responses, and where model systems meet translational constraints.

Against this backdrop, we present the Special Issue entitled “*Molecular advances in oncologic photodynamic therapy*”, comprising six contributions that collectively span mechanistic biology, enabling technologies, and translationally motivated model development. This Special Issue can be organized into three overarching themes: (1) therapy-induced biology and response modulation; (2) targeted and combinatorial PDT strategies; and (3) platform technologies that address long-standing constraints in light delivery, dosimetry, and modeling.

## 1. Theme 1: Therapy-Induced Biology and Response Modulation

A misconception in PDT is that the dominant determinants of outcome are purely photochemical. Two reviews in this Special Issue underscore that PDT is equally a systemic and cellular stressor that reprograms host–tumor interactions in ways that can be either therapeutically enabling or inadvertently tumor-protective.

Domka and colleagues [29] focus on the endocrine dimension of PDT-induced stress signaling, highlighting activation of the hypothalamic–pituitary–adrenal axis and the resulting glucocorticoid surge as a component of the acute-phase response. Beyond PDT-framed descriptive (patho)physiology, the review’s practical implication is that glucocorticoid biology intersects directly with immune cell mobilization, inflammatory modulation, and (context-dependent) cancer cell apoptosis; i.e., variables that are rarely parameterized in PDT protocols but can materially influence post-treatment trajectories. This biological response framework also allows pharmacological modulation to the benefit of therapeutic outcome at the level of the immune system and the inflammatory reaction, which is key for long-term tumor control [10,30,31,32], and cancer cell death, which is instrumental in more acute tumor removal.

Viana Cabral et al. [33] address a second, clinically pervasive dimension of response modulation: chemoresistance and the opportunity for PDT to function not only as a cytotoxic modality, but also as a priming intervention. Photodynamic priming can increase the susceptibility of residual malignancy to subsequent therapies. The review addresses how sublethal photodynamic stress can be operationalized to remodel signaling networks, drug transport, and microenvironmental constraints while also acknowledging limitations that must be confronted for clinical integration, including timing, dosing, and patient-to-patient variability. As widely propagated by our group, sublethal affliction of photosensitized cancer cells (in this case as an element in certain photodynamic priming modalities) could spawn various survival programs that make the cancer cells more recalcitrant to subsequent treatment [34,35].

## 2. Theme 2: Targeted and Combinatorial PDT Strategies

Whereas the intrinsic selectivity of PDT is frequently framed as ‘light confinement’, modern oncological PDT is increasingly defined by molecular selectivity in that targeting vectors, theranostic constructs, and rational combinations have been designed to overcome tumor heterogeneity and multifactorial treatment response pathways [3,4,34,36,37,38,39,40,41,42,43].

Shirke and colleagues [44] provide an elegant empirical exemplar: a prostate-specific membrane antigen (PSMA)-targeted theranostic agent (PSMA-1-MMAE-Pc413) that integrates receptor targeting (PSMA), photodynamic capability (Pc413), and chemotherapy (monomethyl auristatin E; MMAE) into a single construct. Their in vitro data show specificity for PSMA-positive cells and a marked enhancement of light-dependent cytotoxicity relative to single-modality controls. In vivo imaging demonstrates selective tumor uptake; an approach that is mechanistically aligned with the core PDT challenge of achieving high intratumoral PS levels without proportionate off-target exposure.

The contribution by Viana Cabral et al. [33] reinforces a broader principle: the most compelling combination strategies are those that do not merely add modalities, but instead exploit the capacity of PDT to perturb membranes, endolysosomal compartments, vasculature, and immune signaling, thereby sensitizing tumors to orthogonal interventions at the right time and location. In that respect, synergistic modalities (e.g., 1 + 1 = 3) yield a greater net effect than additive modalities (i.e., 1 + 1 = 2) and are hence therapeutically desirable.

## 3. Theme 3: Platform Technologies for Light Delivery, Dosimetry, and Modeling

Two entrenched limitations continue to constrain PDT’s oncological footprint: (i) the finite penetration depth of externally applied light and its implications for the extent of tumor destruction [45,46], and (ii) the difficulty of modeling (and therefore optimizing) PDT in systems that recapitulate clinically relevant tumor microenvironments.

Jia et al. [47] address the first limitation directly via a comprehensive review of laser-free (self-luminescent) photosensitive systems intended to decouple PDT activation from conventional external illumination. Their work encapsulates techniques that circumvent the ramifications of the Beer–Lambert law and instead photochemically confront the cancer cells from within the tumor. The authors map mechanistic classes, including resonance energy transfer, chemically initiated electron exchange luminescence, and Cherenkov radiation energy transfer, and position these strategies as plausible routes to deep-tissue photodynamic activation and imaging while appropriately noting that this field remains in a formative stage with substantial technical caveats and engineering and translational work outstanding.

Balukova et al. [48] address a complementary enabling requirement: the ability to measure and interpret PS behavior in situ. Using TPPS_2a as a model porphyrin-based PS, the authors combine confocal imaging with time-domain fluorescence lifetime imaging microscopy in both 2D monolayers and 3D compressed collagen constructs. The key advance is not merely visual localization, but functional insight: lifetime dynamics report on PS microenvironment- and illumination-dependent changes, which is the type of mechanistic observability needed to move from nominal light dosing toward biologically grounded dosimetry. Although complicated to reduce to clinical practice, this is particularly pertinent in preclinical 3D settings where diffusion barriers and compartmentalization become non-trivial representations of in situ tumors in patients.

Finally, Smith et al. [49] tackle the second entrenched limitation, i.e., tumor modeling, through an extensive review of biomimetic tumor model systems for pancreatic ductal adenocarcinoma (PDAC) in relation to PDT. The authors emphasize PDAC’s desmoplastic stroma, heterogeneity, and therapeutic recalcitrance [50], arguing that more complex models incorporating relevant stromal and immune components are required to develop translatable PDT regimens. Particularly noteworthy is the explicit dismissal of the ‘keep it simple, stupid’ (KISS) principle for PDAC biomimetics. Increasing physiological and biochemical fidelity (e.g., via 3D bioprinting, co-cultures, tumor-on-a-chip platforms, and controlled hypoxia) is framed not as optional sophistication, but as a prerequisite for credible regimen development, especially when immunological recruitment and systemic gradients, absent from most in vitro models, can be decisive for durable tumor control.

## 4. Outlook and Key Frontiers

The six papers in this Special Issue converge on a pragmatic conclusion: the next gains in oncological PDT will not come from incremental changes to any single variable, but from integrated optimization across (patho)biology, photochemistry, targeted PS delivery, and modeling. Several frontiers are particularly salient.

First, response-aware PDT explicitly incorporating stress endocrinology, immune dynamics, survival signaling, and priming effects into regimen design offers a route to more reproducible outcomes, but it also demands that post-PDT biology is regarded as a parameter set to be engineered rather than a by-product to be tolerated [34,51].

Second, molecularly targeted, multi-function constructs can improve tumor specificity and facilitate rational combinations, at least as supported by data generated in animal models [44,52,53,54,55]. However, their promise must be properly contextualized insofar as their efficacy in simplified systems may not be transferable to clinically relevant microenvironments, pharmacokinetics, and host immunity [10,23,56,57,58,59,60,61]. These constitute hurdles that the field of PDT has repeatedly encountered across delivery platforms and that also apply to drug delivery niches beyond PDT.

Third, platform innovation, whether laser-free activation, fluorescence lifetime imaging microscopy-enabled mechanistic dosimetry, or tumor biomimetics of increasing complexity, appears poised to reduce the gap between what PDT can do in principle and what it achieves in practice, although admittedly this progress occurs at the pace of baby steps.

Taken together, the contributions assembled here do not merely catalog advances; they delineate a coherent development path in which mechanistic observability, biologically faithful modeling, and response-modulating combinations jointly enable more translatable PDT as the field moves away from first- and second-generation PS monomodalities. This Special Issue will hopefully stimulate further cross-disciplinary collaboration among molecular oncologists, photochemists and photobiologists, engineers, pharmacologists, and immunologists and accelerate the maturation of oncological PDT into multi-component modalities whose outcomes are more predictable, programmable, effective, and clinically scalable.
